# Study of the effectiveness of a supported intervention package in reducing the risk of avian influenza human exposure through the reduction of infections in poultry: Egypt, 2006–2021

**DOI:** 10.1186/s12985-025-02810-x

**Published:** 2025-05-29

**Authors:** Manal Fahim, Walaa Alim, Shimaa Abukamar, Rabeh El-Shesheny, Wael H. Roshdy, Hossam Hassan, Amira Mohsen, Salma Afifi, Mohamed Abdel Fattah, Radi Hammad, Amr Kandeel

**Affiliations:** 1 Preventive Sector, Ministry of Health and Population, Cairo, Egypt; 2 Centre of Scientific Excellence for Influenza Viruses, National Research Centre, Giza, Egypt; 3 World Health Organization, Egypt Country Office, Cairo, Egypt; 4 The Eastern Mediterranean Public Health Network (EMPHNET), Amman, Jordan; 5 Vice Minister of Health and Population, Ministry of Health and Population, Cairo, Egypt

**Keywords:** Avian influenza, Poultry diseases, Human influenza, Surveillance, Risk reduction

## Abstract

**Introduction:**

For a decade, avian influenza (AI) viruses were major concern for Egypt since they are endemic in poultry and have caused 359 human infections, accounting for 40% of cases globally. Interventions implemented before 2015 proved to have minor impact on the spread of infection. Since 2015, a Supported Intervention Package (SIP) was implemented to reduce the risk of human exposure by reducing infections in poultry. The intervention package included enhanced surveillance and laboratory capacity, early outbreak detection, and raised community awareness. This study aims to evaluate SIP’s effectiveness by comparing number and rates of AI in humans and poultry before and after intervention package implementation.

**Methods:**

AI surveillance data for poultry and humans from 2006 to 2021 was obtained and linked. Human AI data include patients’ demographics, clinical picture, risk factors, lab results and outcome, while poultry data include number prevent of positive specimens for AI by time and place. Confirmation performed by testing oropharyngeal swabs collected from suspected patients and poultry using RT-PCR in the affiliated laboratory. Positive rates were calculated, descriptive data analysis was performed and rate of infection was plotted against demographics and risk factors. Results compared before and after implementation of using Chi^2^ and t-test with *p* < 0.05 significance.

**Results:**

Among all confirmed cases, 346(96.4%) reported before and 13(3.6%) after SIP implementation with no cases reported after 2017. A significant reduction in positivity rate of both human and poultry cases (2.0 vs. 0.2% and 2.4 vs. 1.2%, *p* < 0.001) found after 2015. Percent of housewives decreased from 30.9 to 7.7%, *p* < 0.05 and positive specimens’ rates from backyards decreased from 61.1 to 47.9%, *p* < 0.001. Median days to laboratory confirmation reduced from 3.6 to 2.8 days. The genetic analysis indicated a major genetic drift occurred before 2015, possibly due to inadequate control measures.

**Conclusions:**

The Study indicated reduced infections in humans and poultry suggesting effectiveness of SIP, which also raised community awareness as shown by reducing infections among housewives and enhancing surveillance as shown by case earlier detection. Continued coordinated efforts between human and poultry sectors are needed to contribute to the elimination of the disease in Egypt.

**Supplementary Information:**

The online version contains supplementary material available at 10.1186/s12985-025-02810-x.

## Introduction

Avian influenza virus (AIV) was first identified in birds in the late 19th century and has become increasingly common over time due to many factors, including high-density poultry production, proximity to areas inhabited by wild birds and frequent movement of flocks [1]. The most-known highly pathogenic avian influenza (HPAI) strain, H5N1, was first detected in Scotland poultry outbreaks in 1959 [2]. For several years, the virus haemagglutinin (HA) gene diversified into many genetic groups and multiple genetic lineages. In 1996–1997 the virus caused outbreaks in China and Hong Kong followed by widespread outbreaks in Asia, Africa, the Middle East and Europe in 2003 where it became endemic in many countries [3, 4]. Between 2014 and 2016, HPAI subtypes H5N6 and H5N8 emerged and caused unprecedented mortality in wild birds and poultry in many countries in Africa, Asia, Europe, North America, and Central/South America [5].

Outbreaks of AI are regularly reported in backyard and commercial poultry, and occasionally humans are infected when they come into close contact with infected birds. Many human pandemics of avian influenza have been reported throughout history and in recent years. The human influenza pandemic of 1918 was caused by an H1N1 virus with avian genes and resulted in an estimated 50 million deaths [6]. There have been several outbreaks of AIV in recent years, the largest of which started in Southeast Asia and spread outside the region in 2003 and was caused by H5N1 [7]. Worldwide, 893 confirmed human cases of AI H5N1 and 463 (52%) deaths have been reported to the World Health Organization (WHO) since 2003 till June 2024 [8].

HPAI is a major public health concern in Egypt since it is a vital stopover for millions of migratory birds every year, along with the wide geographical distribution of live poultry market in Egypt, as well as the large amount of poultry production. In Egypt, 80% of all poultry production is carried out in backyards, particularly in rural areas with 5–7 million households raising poultry in their backyards, where raising and selling poultry is the principal income source. HPAI H5N1 was first confirmed in domestic poultry in Egypt in February 2006, when clade 2.2 was isolated from poultry farms and backyards [9]. The virus then underwent substantial genetic evolution with a new clade 2.2.1 becoming dominant specifically in Egypt. The virus then subsequently subdivided into clades 2.2.1.1 and 2.2.1.1a as vaccine-escape mutant and became prevalent between 2009 and 2011 in the commercial sector. In 2014, a drift from the 2.2.1.1 clade resulted in a 2.2.1.2 clade, which gained a gene to enhance binding to the host cell receptor. By the end of 2016, the H5N8 subtype was detected in migratory birds in Egypt. In this regard, Egypt has become an epicenter of the A(H5) virus’ evolution, and there is no clear indication of a resolution [9–11].

The first human case of HPAI H5N1 in Egypt was diagnosed in February 2006. Since then, Egypt has reported 359 human cases (40% of the global total) and 122 deaths (26% of the global total), in addition to 4 human cases of the low pathogenic H9N2 infection reported in 2015–2016 with no cases reported after 2017 [12]. In 2014–2015, Egypt experienced an unprecedented increase in human cases of H5N1 HPAI, probably related to changes in socioeconomic characteristics leading to increased contact between humans and infected poultry or genetic evolution to clade 2.2.1.2, which has a higher affinity to bind human receptors and higher pathogenicity to poultry [9, 10]. Early in the epidemic, Egypt Ministry of Agriculture and Land Reclamation (MOALR) has approved the Animal health livelihood sustainability HPAI strategy to achieve a situation in which H5N1 no longer represents a significant threat to human health. The strategy consisted of three phases namely control phase, consolidation phase, and eradication phase. Despite this, the strategy has not been fully implemented, as the mass vaccination campaigns that initiated to cover all commercial flocks, and backyard poultry have become the only tool to control the H5N1 virus, while other aspects of the control plan have been neglected [9, 13]. In Egypt more than 24 commercial inactivated avian influenza H5 vaccines were licensed for use at poultry farms in Egypt starting 2006. Commercial vaccines showed variable reactivity against earlier antigens, however reactivity declined as the virus mutated. The genetic dissimilarity and poor reactivity of the vaccines indicate that they are ineffective and have led to vaccine-induced escape mutants that have complicated the problem of H5N1 virus circulation in Egypt [11]. ineffective vaccination strategy that failed to control the spread of infection and maintain public health safety was due to lack of adequate funding and communication, an efficient monitoring system, and insufficient training of field workers.These inadequate control measures led to H5N1 virus mutation to occur every year after 2008 when the virus was declared enzootic [13]. The increasing spread, persistence, and viral mutation of AIV among birds raised concern about pandemic potential and posed an ongoing threat to the Egyptian poultry industry.

### Response to the avian influenza 2015 upsurge

During the period October 2014–March 2015, Egypt experienced an increase in H5N1 infections in poultry and humans. Over 400 outbreaks have been reported in commercial and household poultry [14]. The increase in human cases in 2015 in Egypt highlighted the need for a comprehensive strategy to effectively control avian influenza infections in both poultry and humans [9]. In response to the raised concerns, the MOHP supported by WHO and MOALR General Organization of Veterinary Services (GOVS) supported by the United States Agency for International Development (USAID) Global Health Security and Development program, and the Food and Agriculture Organization of the United Nations (FAO) started a supported intervention package (SIP) at national level since 2015 to reduce the risk of human exposure by reducing infections in poultry [15]. The package included:

1. Strengthening surveillance systems for AI in poultry maintained by the MOALR in collaboration with FAO and the World Organization for Animal Health (WOAH). Surveillance includes active, passive, and targeted surveillance systems, which monitor poultry farms, markets and slaughterhouses. Nasal and cloacal swabs and blood samples are collected from birds and tested for AIV Reverse Transcription Polymerase Chain Reaction (RT-PCR) and virus isolation.

Strengthening of the poultry surveillance was conducted through training of the GOVS veterinarians and support staff at central and peripheral levels on surveillance, outbreak investigation and response, and laboratory diagnosis. District-level epidemiological networks in all Egyptian governorates were established and operational to report data from field offices to the GOVS central office through a web-based portal. Active and passive surveillance reporting was enhanced at peripheral and community levels through community animal health outreach, targeted live bird market reporting to enhance surveillance capacity and improve response in the commercial and household poultry production sectors [15, 16].

A Comprehensive strategy for value chain and risk-based surveillance for Avian Influenza and MERS-CoV have been developed in collaboration between GOVS and the FAO Emergency Centre for Transboundary Animal Diseases (ECTAD). The strategy is implemented, maintained and monitored by the GOVS, which oversees all aspects of animal health in Egypt. FAO-ECTAD Egypt continues to provide technical and financial assistance to support the implementation of surveillance in Egypt.

Strengthening of AI surveillance system among humans was carried out through surveillance team sensitization with support from the WHO Partnership Contribution Fund [17]. Case definitions were updated and distributed to all governmental and private health facilities in Egypt. All health facilities were instructed to immediately report suspected AI cases to MOHP using a standardized surveillance form that was distributed to all health facilities. A database was established for suspected and confirmed cases to early detect cases with daily and periodic reports shared with stakeholders to update the epidemiologic situation, contact tracing started immediately upon suspicion for 14 days.

Event-based surveillance (EBS) is essential for the early detection and rapid response to AI, so it has been incorporated into Egypt’s comprehensive surveillance system for AI. Information of Egypt EBS is being collected from a variety of sources including media reports, social media, or reported by people in the community through a hotline or other messaging system. Signals related to AI were added to the list of EBS signals, teams were sensitized to immediate reporting and investigating any human or poultry AI signal. EBS focal points at all levels were instructed to immediately report true AI signals and conduct investigations to actively find any suspected human cases. This enables the identification of unusual patterns or clusters of disease events that could signal AI outbreaks. With real-time or near real-time data, early intervention is made to reduce the spread of the virus among poultry and prevent potential human transmission.

Suspected AI events were added to the list of signals in Egypt Event Based Surveillance (EBS). Teams were sensitized to immediate report and investigate any signal related to AI e.g., diseased or dead poultry including investigation of the human contacts and report to poultry sector.

2- Several laboratories have been established in different governorates and are supervised by a quality management unit at the Central Public Health Laboratory (CPHL) according to a detailed action plan. The laboratories participate in the WHO regional external quality assessment scheme for microbiology and the WHO international external quality assessment program for detection of influenza A virus subtypes by RT-PCR.

The National Laboratory for Quality Control of Poultry Production (NLQP) and the Central Laboratory for Evaluation of Veterinary Biologics (CLEVB) which are two national laboratories of excellence under the GOVS contribute significantly to the diagnosis and control of avian influenza by addressing all circulating AIV subtypes (H5N1, H5N8 and H9N2) in a risk-based approach and the incursion of other subtypes reported in other countries (H7N9, H5N6 and H5N5.), while maintaining early warning of virus spillover and linking diagnosis to rapid response and surveillance of disease control and vaccine use [16]. The project helped to strengthen the laboratory capacity of the NLQP and CLEVB to characterize the genetic and antigenic make-up of currently circulating viruses and to conduct vaccine efficacy trials, as well as disease mitigation and policy advice. Six accredited ISO 17,025 satellite laboratories have been established throughout Egypt in accordance with international standards.

Genomic sequencing plays a critical role in understanding and monitoring avian influenza to track the transmission and evolution of AI and predicting Phenotypes. An effective network of laboratories at governorate level was established to share avian influenza-related laboratory data and genetic material in a timely manner with relevant national and international partners, such as the Global Initiative on Sharing All Influenza Data and GenBank.

Laboratory diagnostic capacity for surveillance and research has been significantly strengthened through in-country and overseas training, provision of equipment, essential consumables and reagents for testing samples collected through active, passive and targeted surveillance activities, and for vaccine evaluation studies. Strengthened capacity for rapid A/H5N1 confirmation and outbreak reporting, achieved through the availability of skilled HPAI diagnostic staff both centrally and in satellite laboratories in the governorates.

3- Early detection of outbreaks was improved through the training of government veterinary clinics staff across Egypt at the village level. These clinics are the first line of detection and treatment for any disease or abnormality based on a specific case definition. Events are recorded in a paper register and sent weekly to the veterinary services in the affiliated district unit, where the data are entered into ECTAD’s sustainable web-based epidemiological network (TADINFO), established through the USAID-funded project to strengthen national capacity for H5N1 surveillance networking between GOVS and local veterinary services. The web-based application facilitates week-by-week comparison of data to detect anomalies in any disease or clinical event in any area. In addition, surveillance of wild birds, which monitors their migratory routes and maintained by MOE was enhanced for early detection of new strains and possible transmission to domestic poultry in Egypt.

4- Biosecurity guidelines and standards have been developed, and training on biosecurity procedures was provided to rapid response teams, field veterinarians and commercial poultry producers. FAO-ECTAD conducted orientation workshops for service providers, veterinarians responsible for on-farm biosecurity surveillance, and a joint workshop to share experiences for poultry producers and vaccination teams.

5- Investigation of risky behavior among human cases was studied in collaboration between MOHP and MOALR epidemiologists guided by the results of surveillance data analysis. Health awareness messages regarding safe poultry handling were developed by a Technical Working Group (TWG) composed of Epidemiology experts at human health and veterinary side. The messages were disseminated to community through more than 14,400 trained MOHP community health workers and CAHO teams.

As part of the Exposure Risk Reduction Program (ERP), a community outreach program to reduce the risk of exposure to HPAI for domestic poultry keepers was carried out. The campaign was designed and implemented to raise public awareness of key effective and protective slaughter practices to reduce the risk of virus transmission from birds to humans during the household slaughter process. Actionable biosecurity messages on safe rearing and slaughtering practices were developed and distributed to farmers and other groups in more than 800 villages. The Community-Based Animal Health Outreach Program (CAHO) was extended to all districts in Egypt’s governorates with more than 250 teams. CAHO teams conducted awareness sessions in health, agricultural, youth and religious centers, schools and veterinary clinics to help communities better understand the importance of these practices.

In the field of coordination and data sharing, a cooperative protocol for data exchange between partners was signed by MOHP, MOALR and MOE. Information on poultry positive foci and updates regarding the type of predominant avian influenza virus is regularly shared with all stakeholders. A media statement includes patient gender, age, governorate of residence and total number of cases and deaths is released as soon as a human AI case confirmed. Effective public-private partnerships by fostering partnerships between the Government of Egypt and private stakeholders such as the Egyptian Poultry Association, pharmaceutical companies, and poultry producers, resulting in the development of the National Strategy for Avian Influenza Control and standard operating procedures for vaccination developed [15]. In addition, a four-way Linking Taskforce comprising representatives from MOHP, GOVS, CPHL and NLQP was established and operationalized to assess health risks at the human-animal interface.

The taskforce collaboration developed the comprehensive HPAI compensation scheme, reconsidering the policy for mass AI vaccination in the domestic poultry sector, and revising the animal health component of the integrated national animal-human preparedness and response plan in Egypt. A sustainability strategy was developed and approved by the MOALR to guide the implementation of longer-term risk reduction measures to control endemic A/H5N1 HPAI in poultry in Egypt [16].

The objective of this study is to describe the changes in avian influenza demographic, epidemiological, and severity of infection in humans in Egypt over time and to correlate those changes with the interventions undertaken on both a human and animal level to assess the impact of the supported efforts made to prevent and control the spread of AIV in Egypt.

### Study methods

Human cases data is collected by interviewing suspected patients identified at any health facility according to national case definition. Suspected case: Any person who shows acute respiratory symptoms with a history of contact with birds within two weeks before the onset of symptoms. Acute respiratory symptoms: Fever greater than or equal to 38 degrees Celsius with one of the following symptoms (cough, shortness of breath, sore throat, muscle and bone pain).History of contact with birds: Exposure/contact with birds (live or dead) whether at (home, bird shop, live bird market, bird farm, travel to an area with positive bird foci).Confirmed case: A suspected case whose positivity has been confirmed by laboratory testing (RT-PCR) for one of the types (H5, H7, H9) performed at the central laboratories of the Ministry of Health (CPHL) or one of the reference laboratories recognized by the World Health Organization.

The surveillance strategy for (AI) relies heavily on laboratory diagnosis, which involves collecting nasopharyngeal and oropharyngeal swabs (NP/OP) from isolated suspected cases, specimens are shipped to the hospital or subnational labs or sent to the Central Public Health Laboratory (CPHL) for (RT-PCR) testing for seasonal influenza viral types A and B. If Influenza A is detected, further testing is performed to determine the subtype, including H1pdm09, H3 former seasonal A(H1N1), highly pathogenic avian influenza A(H5N1) virus, low and highly pathogenic avian influenza A(H7N9) virus, and other subtypes associated with zoonotic events (e.g. H9N2, H7Nx, H5Nx, H10N8), and mixed Influenza infections according to the US Centers for Disease Control (CDC) protocol [9].

Suspected number of poultry with AIV infection, confirmed outbreaks and type of place from where specimens collected was obtained from the poultry surveillance systems.

Different types of surveillance systems for poultry are conducted by MOALR: Active surveillance: is implemented in high-risk governorates where human cases suspected, targeted surveillance among live bird markets (LBMs), in commercial farms, at domestic-wild/migratory bird interface, and passive surveillance through electronic notifications on poultry/human cases from the veterinary clinics, CAHO teams and Hotline established by GOVS for receiving complaints (Woah preparedness plan).

Cloacal and tracheal swabs are collected and tested for all circulating AI sub-types (H5N1, H5N8 and H9N2) and other emerging subtypes reported in other countries (H7N9, H5N6) at the NLQP and CLEVB.

**Fig. 1:**
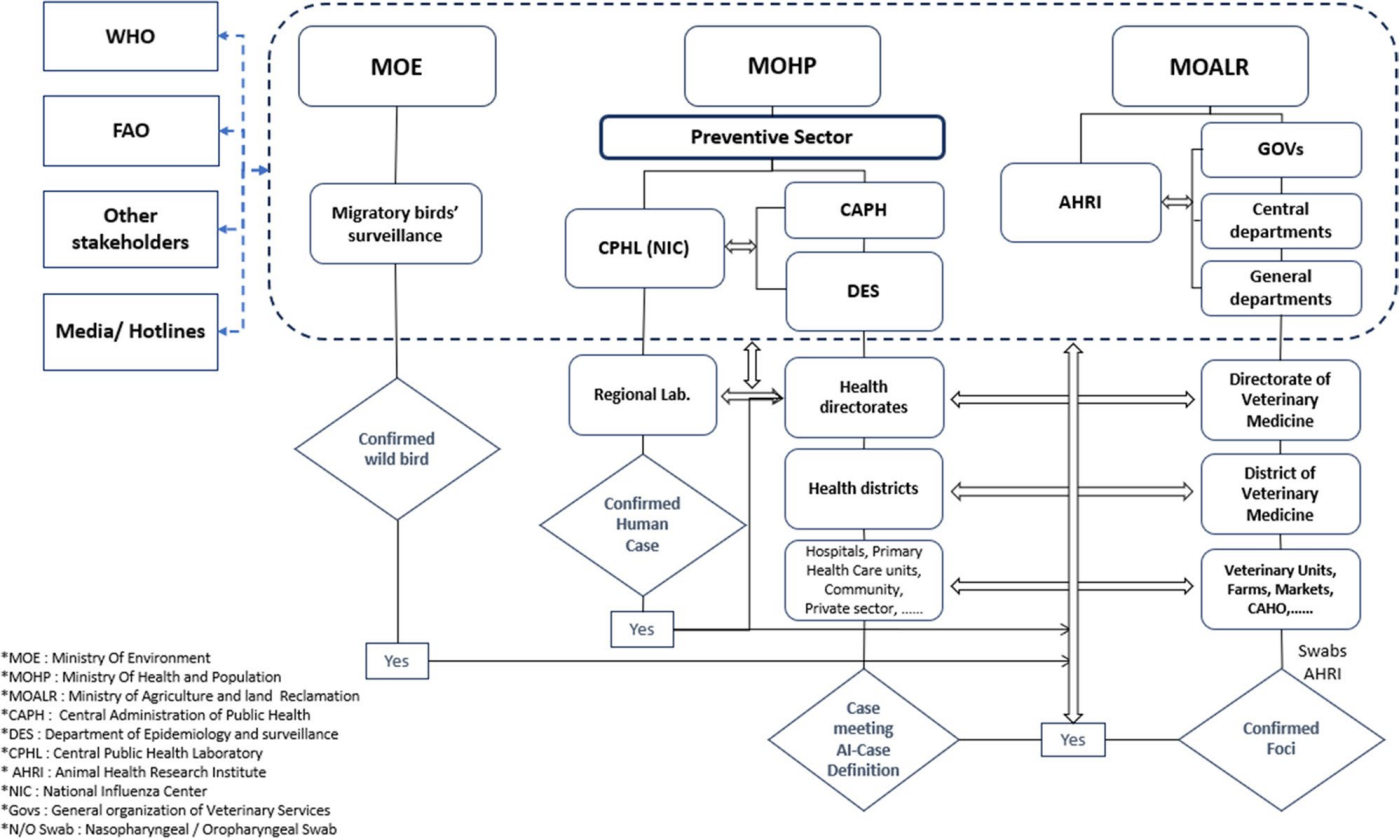
Flow Chart of Avian influenza surveillance and coordination between Organizations in Egypt

Linking between the two datasets was conducted through the collaboration protocol for data exchange between Department of Epidemiology and surveillance at MOHP and (GOVS) under (MOALR) (Fig. 1). When H5N1 AI outbreak occurs, the central Emergency Operation Center (EOC), led by the Prime Minister, is activated, and all relevant ministries are informed to start control measures. Data is shared promptly to enhance response via official documents.

Data of the avian influenza from 2006 to 2021 was obtained. Positivity rates were calculated by year for humans and poultry. Descriptive data analysis was performed for the epidemiology, clinical presentation, and outcome of AI human cases over time. To describe the change before and after implementation of the SIP, characteristics were compared to assess its impact using the Chi2 and t-test with a significance of *p* < 0.05.

Phylogenetic analyses is conducted at CPHL by downloading Egypt HA and NA genes of H5Nx sequences from the Global Initiative for Sharing All Influenza Data (GISAID; https://gisaid.org/). We downloaded all HA sequences of H5 viruses from other host and rejected sequences less than 1500 bp in length, as well as lab-derived sequences, resulting in a total of 529 sequences. Sequences were aligned using the MAFFT [18]. Next clade was used to classify the clades of the sequences and Mega 7 was used for the phylogenetic tree reconstruction by applying the neighbor-joining method with Kimura’s two-parameter distance model and 1000 bootstrap replicates [19, 20]. To identify mutations that might be associated with oseltamivir resistance, the deduced amino acid sequences of H5N1 viruses have been analyzed.

## Results

From 2006 to 2021, of 25,376 AI suspected human cases, 359 (1.4%) were confirmed, their mean age was 20.3 ± 17, with the most affected age groups are children less than five and 15–35 years of age (33.1 and 32.0% respectively) (Tables 1 and 2). Of all cases, 59.3% were females, 44.3% were housewives, 43.5% from Lower Egypt region, and 77.4% occurred during winter to spring seasons, 39.8% had pneumonia, 24.5% admitted to ICU and 34.0% died at hospital. Two peaks were noted, the first was in 2009–2011 and the second in 2014–2015 (Table 2). All of them reported exposure to birds’ exposure to birds two weeks prior to disease onset; of them 338 (94%) exposed to backyard poultry, 15 (4.2%) exposed to live bird markets, 5 (1.4%) were exposed to poultry farms, and only one case (0.3%) exposed to wild birds (Table 2).

**Table 1:** Rate of H5N1 avian influenza positivity among humans and poultry in the period 2006–2021, with a comparison between before and after the implementation of the supported intervention package

Avian influenza infection	Period	No. suspected	Mean No. per year	No. positive	% positive	*P* value
Human	2006–2015	17,465	1,746	346	2.0	< 0.001
	2016–2021	7,911	1,319	13	0.2	
Total human	2006–2021	25,376	1,586	359	1.4	
Poultry	2006–2015	144,370	14,437	3425	2.4	< 0.001
	2016–2021	55,534	9,256	683	1.2	
Total poultry	2006–2021	199,904	12,494	4108	2.1	

**Table 2:** The demographic, epidemiological, and clinical profile of H5N1 avian influenza human cases in the period 2006–2021, with a comparison between before and after the implementation of the supported intervention package

Criteria	2006–2021 (*n* = 359)		2006–2015 (*n* = 346)		2016–2021 (*n* = 13)		*P* value
	No.	%	No.	%	No.	%	
Mean age in years ± SD	20.3 ± 17		20.3 ± 17		20.1 ± 23		0.96
**Age groups in years**							
< 5	119	33.1	114	32.9	5	38.5	< 0.001
5–14	41	11.4	38	11.0	3	23.1	
15–34	115	32.0	114	32.9	1	7.7	
35–55	73	20.3	70	20.2	3	23.1	
> 55	11	3.1	10	2.9	1	7.7	
Gender							
Males	146	40.7	140	40.5	6	46.2	0.69
Females	213	59.3	206	59.5	7	53.8	
Region							
Lower Egypt	156	43.5	155	44.8	1	7.7	0.02
Upper Egypt	134	37.3	125	36.1	9	69.2	
Urban governorates	69	19.2	66	19.1	3	23.1	
**Season**							
Dec-Feb	176	49.0	174	50.3	2	15.4	< 0.01
Mar-May	138	38.4	131	37.9	7	53.8	
Jun-Aug	28	7.8	24	6.9	4	30.8	
Sep-Nov	17	4.7	17	4.9	0	0.0	
Housewives	108	44.3	107	30.9	1	7.7	0.03
Treated with Oseltamivir	178	49.6	167	48.3	11	84.6	< 0.01
Admission-lab confirmation days ± SD	3.6 ± 4		3.6 ± 4		2.8 ± 2		0.21
Admission to discharge Days ± SD	10.0 ± 10		10.0 ± 10		9.7 ± 10		0.91
Duration of illness ± SD	14.3 ± 10		14.2 ± 10		16.8 ± 9		0.35
**Exposures in previous week**							
Exposure to birds	168	46.8	159	46.0	9	69.2	0.06
Breeding poultry at home	104	29.0	99	28.6	5	38.5	0.03
Slaughter poultry	42	11.7	41	11.8	1	7.7	0.04
Clean/prepare poultry	34	19.5	34	9.8	0	0.0	0.13
Exposed to sick birds	34	9.5	33	9.5	1	7.7	0.46
Exposed to dead birds	64	17.8	60	17.3	4	30.8	0.12
Visited farm/store	14	3.9	14	4.0	0	0.0	0.43
**Severity**							
pneumonia	143	39.8	135	39.0	8	61.5	0.06
ICU admission	88	24.5	83	24.0	5	38.5	0.26
Case fatality	122	34.0	117	33.8	5	38.5	0.36

Among all confirmed cases, 346 (96.4%) reported during the first ten years (2006–2015) before the implementation of the SIP and 13 (3.6%) reported after its implementation with no cases reported after 2017. Between the two periods, there was a shift of the age group from middle age to primary school age (Middle age decreased from 32.9 to 7.7% and school age increased from 11.0 to 23.1%, p = < 0.001), percent of females decreased from 59.5 to 53.8%, *p* = 0.69, and the percent of housewives decreased from 30.9 to 7.7%, *p* = 0.03. By region, there was a shift from Lower Egypt to Upper Egypt (Lower Egypt from 44.8 to 7.7% and Upper Egypt from 36.1 to 69.2%, *p* < 0.01) and by season there was a shift from winter-spring to spring-summer (Table 2).

The mean days to lab confirmation decreased from 3.6 ± 4 to 2.8 ± 2 and hospital days from 10.0 ± 10 to 9.7 ± 10, and duration of illness increased from 14.2 ± 10 to 16.8 ± 9 (Table 2).

Severity of illness increased between the two periods in terms of pneumonia (39. Vs 61.5%, *p* = 0.06), ICU admission (24.0 vs. 38.5%) and case fatality (33.8 vs. 38.5%) but not statistically significant.

Percent exposed to birds increased from 46.0 to 69.2%, backyard breeding 28.6–38.5%, and dead birds 17.3–30.8%, while percent participated in slaughtering, preparing, exposure to sick birds and visit poultry market or store decreased (11.8 to 7.7% and 9.8 to 0.0% and 9.5 to 7.7% and 4.0 to 0.0% respectively) (Table 2).

**Fig. 2:**
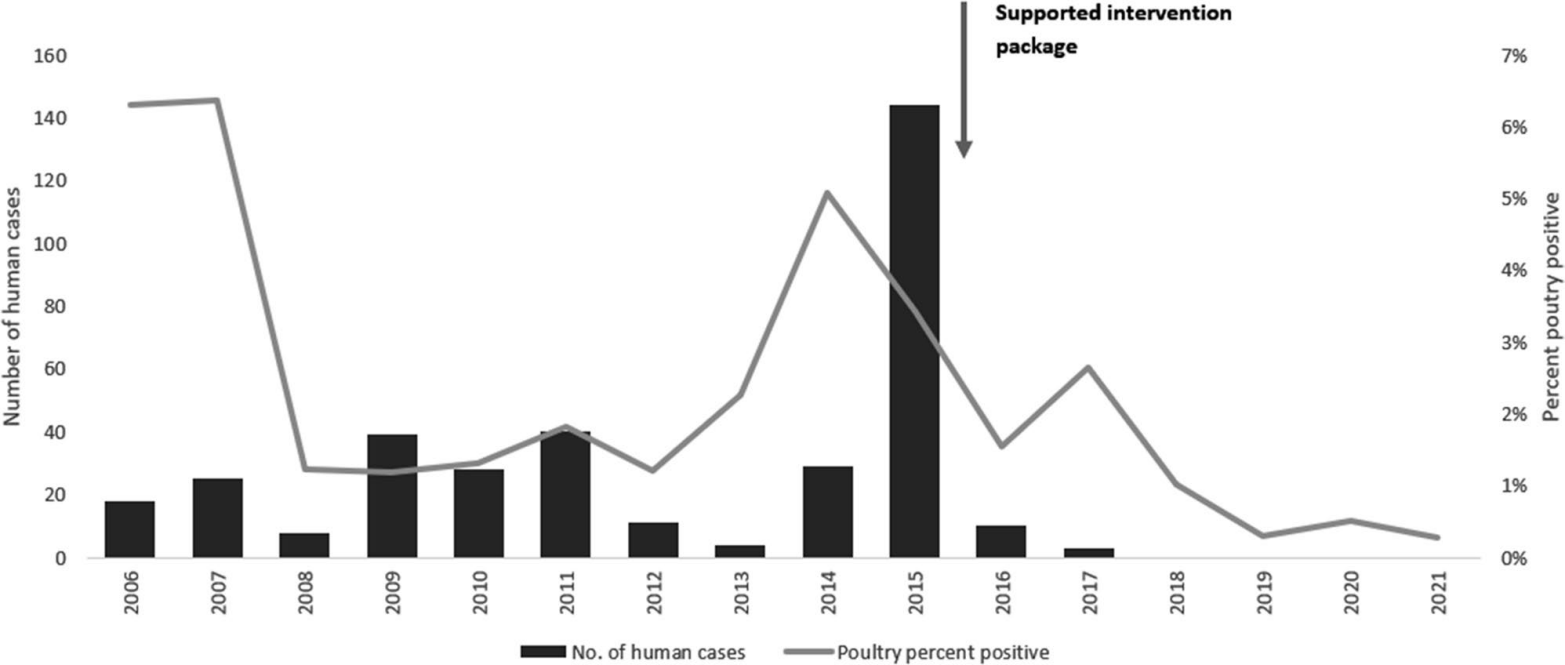
Number of human H5N1 avian influenza cases and rate of positivity in poultry, 2006-2021

**Fig. 3:**
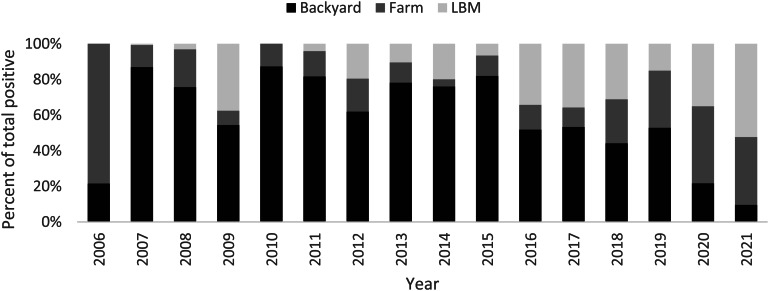
Distribution of H5N1 avian influenza poultry outbreaks by place of specimen collection, 2006-2021. *Adapted and modified from Self-declaration of freedom from High pathogenicity avian influenza (with and without vaccination) in poultry compartments in Egypt. Available at: https://www.woah.org/fileadmin/Home/eng/Animal_Health_in_the_World/docs/pdf/Self-declarations/Annexes/Egypt_AI_Annex_8.pdf

**Fig. 4:**
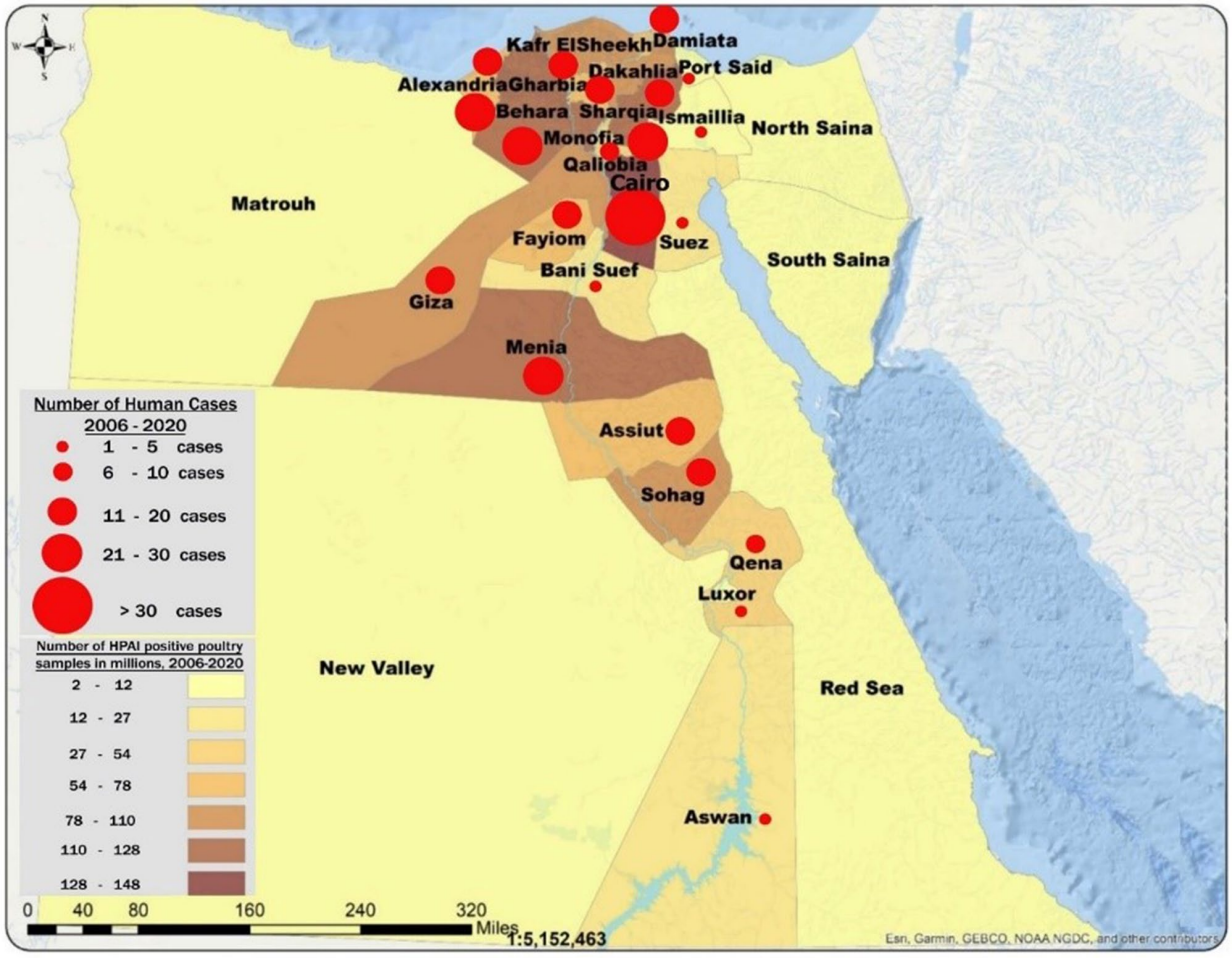
Distribution of confirmed H5N1 human cases, and special distribution of HPAI positive poultry samples, 2006–2020. * Adapted and modified from Highly Pathogenic Avian Influenza IN EGYPT,16th conference of WOAH regional commission for the Middle East, November 2021. Available at: https://rr-middleeast.woah.org/app/uploads/2021/11/4-highly-pathogenic-avian-influenza-2021-egypt.pdf

Increased detection of poultry foci coincides with the surge of human cases especially during 2014–2015 (Fig. 2). The rate of positivity in both poultry and human specimens decreased significantly before and after 2015 (2.4 to 1.2% and 2.0 to 0.2%, *p* < 0.001 respectively) and the percentage of backyard positive specimens among positive poultry specimens declined significantly from 61.1 to 47.9%, *p* < 0.001 (Fig. 3). Figure 4 shows that most of the governorates with higher rates of poultry AI outbreaks are having high number of cases, and overall Lower Egypt has higher poultry positivity rate and number of human cases.

**Fig. 5:**
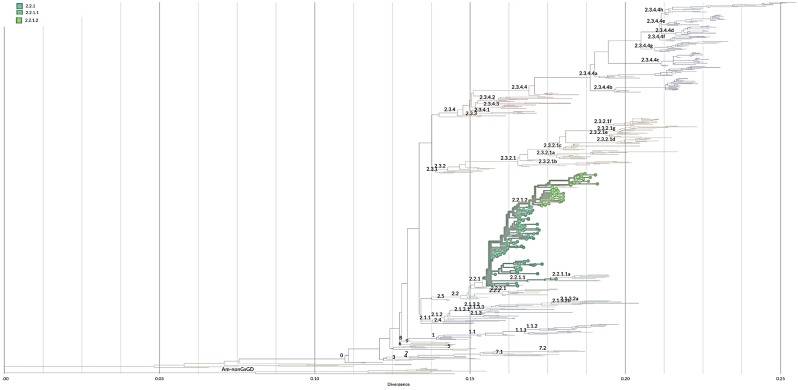
Phylogenetic tree of HA gene of H5N1 virus sequenced from human in Egypt. Tree was generated by https://clades.nextstrain.org, branches are colored according to H5 clades, circle dote represent the sequences of human viruses that belong into three clade: 2.2.1, 2.2.1.1, and 2.2.1.2

Phylogenetic analysis of the H5 hemagglutinin (HA) was conducted to determine the HA clades of 148 viruses sequenced from humans in Egypt (Fig. 5). All viruses were found to share a common ancestor with clade 2.2 viruses.

H5Nx viruses in Egypt comprised five clades: 2.2.1, 2.2.1.1, 2.2.2.1.1a, 2.2.1.2 and 2.3.4.4b (Figure S1), human viruses belong into three clade: 2.2.1, 2.2.1.1, and 2.2.1.2 (Fig. 5). The majority of the viruses clustered into clade 2.2.1 sequenced from 2006 to 2011 and the viruses sequenced between 2011 and 2015 belonged to clade 2.2.1.2 and only one strain belonged to clade 2.2.1.1 (figure S1).

The analysis of the NA gene of human H5N1 viruses revealed that none of these viruses displayed oseltamivir resistance markers E119, H275, R293 and N295 (N1 numbering) except three viruses had mutation at position N295S belong to clade 2.2.1 (A/Egypt/14724-NAMRU3/2006, A/Egypt/14725-NAMRU3/2006, and A/Egypt/N11981/2009).

## Discussion

Scientists generally believe that influenza strains will be responsible for the next global pandemic of deadly infectious diseases in the near future [20]. The diversity of zoonotic influenza viruses that have recently caused human infections is a cause for concern. Recently, WHO was notified of a serious incident involving the first human case of A(H5N1) virus infection after exposure to cows believed to be infected with A(H5N1) [17]. In the current situation, it is essential to integrate surveillance systems among animal, environment (wild birds) and human populations and to study changes in AIV and the effectiveness of different interventions.

Different studies evaluated different interventions implemented by countries the control AI however no studies have evaluated a package of interventions [21–24]. This study evaluates a package of different interventions to control AI in humans and poultry. It revealed a significant decline in AIV rate of positivity in poultry and number of human cases with no cases reported after 2017. This decline coincides with the implementation of SIP conducted in collaboration between MOALR, MOHP and FAO and funded by USAID to reduce the risk of human exposure by reducing infections in poultry [15, 16]. This reduction could have occurred due to multi factors. One factor could be the effectiveness of the supported plan of interventions including enhanced surveillance, improve public awareness and safe practice, develop biosecurity, and enhance collaboration between partners. The dramatic reduction in the positivity rate among poultry supports this possibility where the increase in infections among poultry was always followed by a wave of increased number of human cases. Moreover, the reduction in the percent of human cases exposed to slaughtering or preparing poultry and reduction in percent of positive specimens collected from backyards could indicate the successfulness of the community outreach campaigns [16]. Another factor could be the predominance of H5N8 which dramatically reduced H5N1 virus in poultry [9]. Studies suggested that the risk of infection with H5N8 virus in the general population is very low [25].

This study provides evidence of the effectiveness of the intervention package. First is the shift in the most at risk groups from middle aged housewives to children < 15 years could indicate the success of the community outreach campaigns that targeted female backyard breeders [20]. In an investigation of risky behavior related to handling poultry, it was discovered that children are usually watching the poultry culling and slaughtering process (MOHP communication). Awareness campaigns should be extended to primary school children and their mothers to reduce their exposure to poultry.

Another evidence is the reduction in the time to laboratory confirmation. This may indicate the success of raising laboratory staff capacity in terms of training and capacity building.

Another evidence of the effectiveness of the outreach community education campaigns in teaching the community about proper poultry handling is the decrease in human cases involved in slaughtering or preparing poultry, as well as the reduction in the number of positive poultry specimens collected from backyards. As a result of the increase in exposure to dead poultry, education about the proper disposal of dead birds may be necessary.

A positive geographical correlation between poultry outbreaks and human cases was demonstrated in the study, which provided evidence of the usefulness of collaboration between the MOHP and the MOALR in the exchange of data. The shift in the region and season of AIV human infections after 2015 found in this study may be due to different levels of implementation of the intervention package, changes in environmental factors (such as poultry density or annual precipitation), or changes in the geographical and temporal distribution of the poultry epizootics [26, 27].

The study suggests that the severity of human infection increased after 2015 in terms of higher ICU admissions, case fatalities, and longer illness duration. An increase in disease severity may be related to delay of health care seeking behavior and deny to report poultry exposure. There is a need for further research into the change in resistance to oseltamivir as well as increased awareness of AIV in the community and among physicians.

The genetic analysis in this study showed a major genetic drift occurred after 2011 possibly due to ineffective poultry vaccine policy and inadequate control measures. Sequencing proved that the viruses that infected humans had the same genetic composition of the viruses described in poultry in the same period of time in accordance with previous study conducted on human and poultry isolated from Egypt [11]. This has highlighted the ineffectiveness of control measures implemented prior to 2015 and their impact on human AI infections. The effective vaccination strategy adopted by China in the poultry industry has been successful in reducing the incidence of H5N1 in poultry. In order for the vaccination strategy to be successful, the vaccine seed virus must be updated to ensure the best possible protection against the prevalent circulating strain. As a key component of this strategy’s success is that the vaccine seed virus is updated in order to ensure maximum protection against the most prevalent strain of the virus [28]. This highlights the importance of monitoring the circulating strains for developing effective vaccines and to detect viral mutations early and predict their role in human infections.

Regular monitoring of genetic changes in the AI virus is recommended to detect viral mutations early and predict their role in human infections.

## Conclusions

The study showed a significant reduction in the number of human AIV infections and poultry outbreaks, partly due to the supported integrated intervention plan implemented in 2015–2020. The study proved the effectiveness of the strengthening of laboratory capacity and community education campaign on safe poultry handling. There is a need to raise awareness among the community for proper disposal of dead birds. Integration of the AIV human, animal and laboratory data has contributed to better understanding of the disease epidemiology and clinical picture. Maintaining surveillance of avian influenza in animals and humans and data sharing are essential for guiding early case detection and hence control of outbreaks. Maintaining raising of awareness for healthcare workers to early suspect cases and for patients to seek medical advice early should be continued. To maintain control of avian influenza in humans in Egypt, it is essential to maintain and coordinate surveillance systems to ensure early detection and rapid response to outbreaks and monitoring of virus evolution are vital to assess the effectiveness of interventions. Public awareness campaigns should educate communities about the risks of the disease and safe practices for handling poultry. Vaccination programs along with strict biosecurity measures in poultry farms and markets should be enhanced to reduce human exposure to infected birds. Regulating live poultry markets with improved hygiene standards or closures where necessary will further minimize risk. International collaboration with global health organizations can bolster data sharing, resource allocation, and strategy development.

### Study limitations

This study has three limitations, one is the small number of human cases after the implementation of the SIP, which decreases the significance of comparisons. Second, the study did not examine the AIV infection in wild birds to obtain a more accurate picture of the overall situation in Egypt pertaining to avian influenza. In addition, other factors such as natural attenuation of H5N1 virus, competitive substitution by H5N8, and environmental changes may also contribute to the reduction of human cases. However, the control measures done were more likely to succeed than other factors because they were systematic and effective approach to disease control.

## Electronic supplementary material

Below is the link to the electronic supplementary material.

Supplementary Material 1

## Data Availability

No datasets were generated or analysed during the current study.
