# Supplementary figures and images for: Study of the effectiveness of a supported intervention package in reducing the risk of avian influenza human exposure through the reduction of infections in poultry: Egypt, 2006–2021
Source: Virol J. 2025 May 29;22:170. doi: 10.1186/s12985-025-02810-x (PMC12123743; doi:10.1186/s12985-025-02810-x)

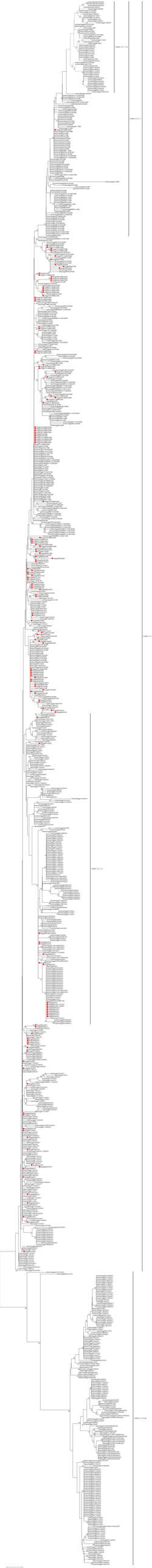

Supplement: Supplementary file 1 — Supplementary Material 1 [file 12985_2025_2810_MOESM1_ESM.pdf]
